# Proton Therapy of Prostate and Pelvic Lymph Nodes for High Risk Prostate Cancer: Acute Toxicity

**DOI:** 10.14338/IJPT-20-00094.1

**Published:** 2021-09-14

**Authors:** Richard Choo, David W. Hillman, Thomas Daniels, Carlos Vargas, Jean Claude Rwigema, Kimberly Corbin, Sameer Keole, Sujay Vora, Kenneth Merrell, Bradley Stish, Thomas Pisansky, Brian Davis, Adam Amundson, William Wong

**Affiliations:** 1 Department of Radiation Oncology, Mayo Clinic, Rochester, MN, USA; 2 Department of Biomedical Statistics and Informatics, Mayo Clinic, Rochester, MN, USA; 3 Department of Radiation Oncology, Mayo Clinic, Scottsdale, AZ, USA

**Keywords:** prostate cancer, proton therapy, hypofractionation, acute toxicity

## Abstract

**Purpose:**

To assess acute gastrointestinal (GI) and genitourinary (GU) toxicities of intensity-modulated proton therapy (IMPT) targeting the prostate/seminal vesicles and pelvic lymph nodes for prostate cancer.

**Materials and Methods:**

A prospective study (ClinicalTrials.gov: NCT02874014), evaluating moderately hypofractionated IMPT for high-risk or unfavorable intermediate-risk prostate cancer, accrued a target sample size of 56 patients. The prostate/seminal vesicles and pelvic lymph nodes were treated simultaneously with 6750 and 4500 centigray radiobiologic equivalent (cGyRBE), respectively, in 25 daily fractions. All received androgen-deprivation therapy. Acute GI and GU toxicities were prospectively assessed from 7 GI and 9 GU categories of the Common Terminology Criteria for Adverse Events (version 4), at baseline, weekly during radiotherapy, and 3-month after radiotherapy. Fisher exact tests were used for comparisons of categorical data.

**Results:**

Median age was 75 years. Median follow-up was 25 months. Fifty-five patients were available for acute toxicity assessment. Sixty-two percent and 2%, respectively, experienced acute grade 1 and 2 GI toxicity. Grade 2 GI toxicity was proctitis. Sixty-five percent and 35%, respectively, had acute grade 1 and 2 GU toxicity. The 3 most frequent grade 2 GU toxicities were urinary frequency, urgency, and obstructive symptoms. None had acute grade ≥ 3 GI or GU toxicity. The presence of baseline GI and GU symptoms was associated with a greater likelihood of experiencing acute GI and GU toxicity, respectively. Of 45 patients with baseline GU symptoms, 44% experienced acute grade 2 GU toxicity, compared with only 10% among 10 with no baseline GU symptoms (*P* = 0.07). Although acute grade 1 and 2 GI and GU toxicities were common during radiotherapy, most resolved at 3 months after radiotherapy.

**Conclusion:**

A moderately hypofractionated IMPT targeting the prostate/seminal vesicles and regional pelvic lymph nodes was well tolerated with no acute grade ≥ 3 GI or GU toxicity. Patients with baseline GU symptoms had a higher rate of acute grade 2 GU toxicity.

## Introduction

External beam radiotherapy (RT) has been a mainstay for the treatment of high-risk, clinically localized prostate carcinoma (PC). In high-risk PC, the risk of occult pelvic nodal metastasis can be considerable. Most large randomized trials demonstrating the benefit of RT or of adding androgen-deprivation therapy (ADT) to RT for high-risk PC have encompassed the regional pelvic nodes as part of clinical target volumes (CTVs) of RT [1–6].

The standard RT technique for prostate cancer in most centers is intensity-modulated RT (IMRT). In recent years, intensity-modulated proton therapy (IMPT) has become more widely available for the treatment of PC. Although IMRT provides a conformal dose distribution to CTVs, it is achieved by spreading out the integral dose over a large volume of healthy tissues with a considerable dose to organs at risk (OARs). Protons, in contrast to photons, deposit most of their energy at a depth in accordance to their energy, resulting in a distinct dose distribution with negligible exit dose. Thus, IMPT can offer an additional benefit over IMRT in sparing nontarget tissue while maintaining conformal dose coverage to CTVs. This dosimetric advantage of IMPT can be amplified, when CTVs expand over a large anatomical area that poses a larger volume of nearby OARs to unnecessary radiation exposure. Such an example is pelvic nodal irradiation; in which, IMPT can provide substantial sparing of nontargeted pelvic organs, such as the bladder, rectum, large bowel, and small bowel, in comparison with IMRT. This dose reduction to OARs can then translate into a decrease in gastrointestinal (GI) and genitourinary (GU) toxicity.

Moreover, PC has a very low α/β ratio (1.4-3) [7–10]. The low α/β ratio of PC implies that a hypofractionated regimen with a larger than conventional dose per fraction can be more effective in eradicating PC. Furthermore, if the α/β ratio of PC is less than that of the adjacent OARs (eg, rectum and bladder, α/β ratio = 3-5), a hypofractionated regimen can provide a therapeutic gain, either by improving the killing of tumor cells while keeping the risk of radiation toxicity to OARs same or by reducing the risk of radiation toxicity to OARs while delivering an isoeffective dose to the PC. Recently, several randomized studies have shown that a moderately hypofractionated regimen (2.5-3.4 Gy per fraction given over 4-6 weeks) can be equally efficacious and as safe as a conventionally fractionated regimen (1.8-2 Gy per fraction given over approximately 8 weeks) for PC [11–15]. As a result, a moderately hypofractionated regimen has been increasingly accepted in routine practice.

In current clinical practice, proton beam therapy for PC has been mainly used when CTVs are limited to the prostate and the seminal vesicles. When CTVs are expanded to include the regional pelvic lymph nodes, along with the prostate/seminal vesicles, the data on the use or efficacy of proton beam therapy have been very limited. There are even fewer data on the use of proton beam therapy combined with a hypofractionated regimen that treats both the prostate/seminal vesicles and pelvic lymph nodes.

A study of proton beam therapy with a moderately hypofractionated regimen for patients with high- or unfavorable intermediate-risk prostate cancer has completed accrual at our institution. In this study, IMPT was used to deliver a hypofractionated radical dose to the prostate and seminal vesicles, while simultaneously delivering a conventionally fractionated dose to the regional pelvic lymph nodes over the same total number of fractions. The primary objective of the study was to evaluate the acute and late toxicity of the study regimen.

The aim of this article is to report the incidence and severity of acute GI and GU toxicity of this moderately hypofractionated proton beam therapy targeting the regional pelvic lymph nodes and the prostate/seminal vesicles, using prospectively collected toxicity data.

## Materials and Methods

A prospective study of proton beam therapy with a moderately hypofractionated regimen is in progress for patients with high- or unfavorable intermediate-risk prostate cancer since August 2016. The study was registered at clinicaltrials.gov (ClinicalTrials.gov Identifier: NCT02874014) [16] and was approved by our institutional review board. In the study, the prostate and the seminal vesicles (CTV-high) were treated with a hypofractionated regimen using daily 2.7-Gy fractions to 67.5 Gy over 5 weeks, whereas the regional pelvic lymph nodes (CTV-low) were simultaneously treated with daily 1.8-Gy fractions to 45 Gy. Assuming that the α/β ratio value of PC is in the range of 1.5 to 3, 67.5 Gy in 2.7 Gy fractions is equivalent to 80.2 Gy to 85.9 Gy in 1.8 Gy fractions.

Eligible patients had histologically confirmed adenocarcinoma of the prostate with at least one of the following high-risk features: clinical stage T3-4, Gleason score ≥ 8, or prostate-specific antigen (PSA) > 20 but < 100 ng/mL. Patients with unfavorable intermediate prostate carcinoma (T1-2, Gleason score 4 + 3, and PSA 10-20 ng/mL) were also eligible. Patients with distant or pelvic lymph node metastasis were ineligible for the study. Staging workup included a bone scan and a computed tomography (CT) scan or magnetic resonance imaging scan of the abdomen and pelvis.

For the sample size calculation of the study, it was hypothesized that ≤ 5% of patients would encounter late-grade ≥ 3 GI or GU toxicity after 2 years of follow-up. The proposed treatment strategy would be considered unacceptable, if late-grade ≥ 3 GI or GU toxicity was ≥ 15%. A sample size of 51 evaluable patients was needed, using a 1-stage binomial design, to test the null hypothesis that the rate of late-grade ≥ 3 GI or GU toxicity is ≥ 15%. The target sample size was increased to 56 patients to account for cancellation, major treatment violation, and lost follow-up. The study completed the target accrual of 56 patients in December 2018.

As part of the standard of care for high- or unfavorable intermediate-risk PC, patients also received ADT for a duration of 4-36 months. It started 2 months before the beginning of RT, and consisted of a luteinizing hormone-releasing hormone agonist (goserelin or leuprolide) plus bicalutamide 50 mg by mouth once daily for 2-4 weeks, given at the start of ADT.

The manner that the proton beam therapy was prepared (such as simulation, definition of CTVs, and treatment planning) and delivered for the clinical study was described in our previously published work [17].

### Simulation

A minimum of 1 day before a scheduled CT simulation, 4 carbon markers were implanted into the prostate gland via a transperineal or transrectal approach under transrectal ultrasound guidance. These implanted carbon markers were used for daily image-guided proton beam therapy. No rectal balloon or hydrogel spacer was used in the study.

Patients had CT simulation with a full bladder. Patients were also instructed to have a bowel movement in the morning and to use a restroom 60 minutes before simulation to evacuate any residual bowel gas or stool. A planning CT scan was performed with the patient in the supine position. An indexed knee cushion and a custom vacuum-lock bag were used to immobilize the legs and feet. The CT images were obtained from above the iliac crests to the mid femur with 2-mm slice thickness. Shortly after CT simulation was completed, a magnetic imaging resonance scan of the pelvis (an MR simulation) was obtained with the same setup.

### Volume Definition

Both CT and MR images were imported into the Eclipse treatment planning system (Varian Medical Systems, Palo Alto, California), and the 2 data sets were co-registered, using the implanted intraprostatic carbon markers as references.

The CTVs were delineated on CT images, with MR images used to aid delineating CTV-high. The CTVs were defined in accordance to the consensus guidelines of the Radiation Therapy Oncology Group (RTOG) [18]. The CTV-high comprised the prostate and the seminal vesicles. The extent of the seminal vesicles to be included in CTV-high was at the discretion of the attending physician and was based on clinical and pathologic features of disease. The CTV-low encompassed the regional pelvic lymph nodes. It included the obturator, external iliac, proximal internal iliac, distal common iliac (up to a level corresponding to the sacral promontory or L5-S1 junction), and the presacral nodes (extending inferiorly to S3, depending on whether the dose constraints to the rectum were achievable). The CTV-low was delineated with a 7-mm margin in 3 dimensions to the iliac vessels and a 10-mm margin anteriorly from the anterior sacral bone for presacral nodes. Adjacent healthy organs (such as the rectum, small bowel, large bowel, and bladder), pelvic musculature, and bones were carved out from CTV-low. The pelvic organs at risk (OARs) were contoured, using the RTOG guidelines, and included the rectum, large bowel, small bowel, bladder, penile bulb, and the femoral heads. The planning target volume (PTV)-high was derived by 5-mm expansion around CTV-high, except 4-mm expansion in the posterior direction. The PTV-low was obtained by 5-mm expansion around CTV-low.

### Treatment Planning

The IMPT was prepared with predefined dose-volume histogram (DVH) objectives for target volumes and OARs. Quantitative evaluation of plans was performed by means of a standardized DVH. For CTVs and PTVs, D98% and D2% (dose received by ≥ 98% and 2% of the volume) were evaluated as metrics for maximum and minimum doses, along with V100%, and V107% (the volume receiving ≥ 100%, and ≥ 107% of the prescribed dose). For OARs, mean dose, maximum dose expressed as D2cc (Gy), and a set of appropriate volume metrics were examined. For the bladder, DVH objectives were D2 cm^3^ < 72.9 Gy, V66 Gy < 8%, V61 Gy < 11%, V57 Gy < 15%, and V36 Gy < 33%. Rectum DVH objectives were D2 cm^3^ < 71.5 Gy, V66 Gy < 9%, V61 Gy < 12%, V 57 Gy < 15%, V53 Gy < 17%, and V44 Gy < 24%. Small-bowel DVH objectives were a maximum of < 52 Gy, V50 Gy < 2 cm^3^, V45 Gy < 150 cm^3^, and V30 Gy < 300 cm^3^.

The Eclipse treatment planning system was used to generate an optimized proton plan. The planning process involved the inverse optimization of dose distribution generated by a number of pencil beam spots scanned for cloud-covering targets and OARs and the modulation of each beam spot with simultaneous tuning of spot energy and weight. Spot spacing was set to 3 mm. Gantry angles were individually chosen for the best geometric setting for an individual patient's anatomy and target configuration. Two opposed lateral beams were most commonly used. Plan quality and acceptability were assessed, based on DVH parameters of target volumes and OARs.

### Daily Proton Therapy

Proton therapy was delivered with daily matching of the prostate position, which was achieved by on-line matching of the intraprostatic carbon markers with the onboard orthogonal-kV imaging.

When proton therapy was delivered with a daily setup based on the prostate position, there was uncertainty about the dose coverage of the regional pelvic nodes whose position can be independent of prostate motion. To assess the adequacy of the CTV coverage during the 5-week course of proton therapy, a verification the CT scan was obtained weekly, starting 1 day before the start of the proton therapy. The CTV-high and CTV-low were propagated from the planning CT scan onto the verification CT scans by matching intraprostatic carbon markers and pelvic bones, respectively. Coverage of CTVs was then evaluated on the weekly verification CT scans. When the coverage of CTVs was found inadequate, a new IMPT plan was generated, implemented, and evaluated with weekly verification CT scans.

### Toxicity Evaluation

Baseline GI, GU, and erectile function were collected before RT, using the National Cancer Institute Common Terminology Criteria for Adverse Events, version 4 (CTCAE; NCI, Bethesda, Maryland). In addition, other questionnaires, including EPIC-26 (the Expanded Prostate Cancer Index Composite–26), the Patient-Reported Outcomes version of the CTCAE, the AUA (American Urological Association) Symptom Index score, and the erectile domain of the International Index of Erectile Function were administered.

Seven GI categories from the CTCAE were used to assess GI toxicity: diarrhea, fecal incontinence, proctitis, rectal hemorrhage, rectal stenosis, rectal ulcer, and small-intestinal obstruction. For the assessment of GU toxicity, 9 GU categories were used: cystitis (noninfective), urinary frequency, urinary urgency, urinary tract obstruction, urinary retention, urinary tract pain, bladder spasm, urinary incontinence, and hematuria. Erectile dysfunction category was used for the evaluation of erectile function

All patients were assessed weekly during RT with the documentation of treatment tolerance and any GI and GU toxicity. Grade ≥ 3 acute toxicity was considered to be a significant, acute, treatment-related toxicity. Toxicity assessment was also administered at the end of RT, and at 3, 6, and 12 months after RT, followed by every 6 months up to 60 months after RT. Acute toxicity included the side effects experienced up to 3-month after RT. Late toxicity was defined as toxicity persisting for > 3 months or developing > 3 months after RT. An attending physician or his or her clinical assistant administered the toxicity questionnaire and recorded a toxicity grade. The recorded toxicity grades were entered into the database, and that prospectively collected database was the basis for the toxicity analysis.

The toxicity data were analyzed as of September 2019; by which point, all patients had completed the planned proton beam therapy and had ≥ 3-month post-RT follow-up. The details of other outcomes, including quality of life, late toxicity, and PSA relapse-free survival, are beyond the scope of this report and will be reported separately. One patient was excluded from the acute toxicity analysis. That patient died of cardiovascular disease before the start of RT, but after completing the initial study registration. Thus, the data from 55 patients were available for the acute-toxicity analysis.

### Statistical Analysis

For acute toxicity, the maximal toxicity grade achieved was evaluated. In addition, the proportion of patients scoring grade ≥ 1 toxicity at baseline, during RT, and at 3 months after RT was calculated. Fisher exact tests were used for comparisons of categorical data.

## Results

### Patient Characteristics

Between August 2016 and December 2018, a total of 55 patients completed the planned proton therapy. The characteristics of these patients are summarized in **Table 1**. Median age was 75 years (range, 55-87 years). All patients had good performance status (ECOG 0, 48 patients; ECOG 1, 7 patients). Median pretreatment PSA was 10.24 ng/mL (range, 0.65-97.3 ng/mL). Median Gleason score was 8. Fifty-two patients had a high-risk prostate carcinoma; 3 had an unfavorable intermediate-risk prostate carcinoma. All received ADT with a median duration of 18 months (range, 4-37 months).

**Table 1.** Patient baseline characteristics (n = 55).

**Parameter**	**Value**
Age, y	
Mean	74.3
Median	75
Range	55–87
Gleason score, No. of patients (%)	6, 5 (9)
	7, 17 (31)
	8–10, 33 (60)
Baseline PSA, ng/mL	
Mean	18.8
Median	10.24
Range	0.65–97.3
T stage, No. (%)	
T1–T2	23 (42)
T3a	22 (40)
T3b	10 (18)
Risk category, No. (%)	
High risk	52 (95)
Unfavorable intermediate risk	3 (5)
Duration of ADT, months	
Mean	17.1
Median	18
Range	4–37

**Abbreviations:** PSA, prostate-specific antigen; ADT, androgen-deprivation therapy.

### Assessment of Tolerability and Acute Treatment-Related Toxicity

Median follow-up was 25 months (range, 5-40 months). All completed the planned proton beam therapy without major treatment interruption. None experienced treatment interruption or delay for ≥ 3 days. Median overall treatment duration was 34 days (range, 32-37 days).

**Figure 1.**
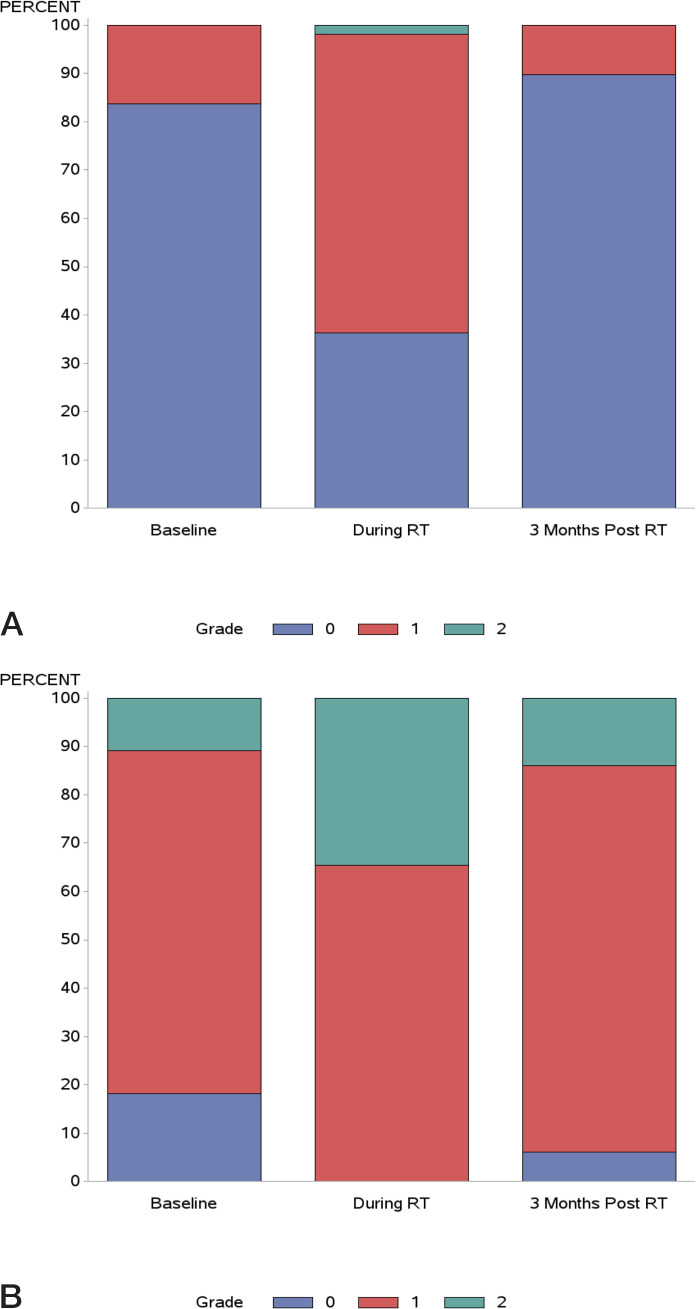
(A) Baseline gastrointestinal (GI) symptoms and maximum acute GI toxicity scores in the 7 GI categories during radiotherapy (RT) and at 3 months after RT. (B) Baseline genitourinary (GU) symptoms and maximum acute GU toxicity scores in the 9 GU categories during RT and at 3 months after RT.

Baseline GI symptom and acute GI toxicity up to 3 months after RT are summarized in **Figure 1A**. At baseline, most patients (84%: 46 of 55) had no GI symptoms. During RT, grade 1 and 2 GI toxicity was 62% and 2%, respectively; grade 2 GI toxicity was proctitis occurring in 1 patient (2%). At 3 months after RT, grade 1 and 2 GI toxicity declined to 9% and 0%, respectively. None experienced ≥ grade 3 acute GI toxicity.

Baseline GU symptom and acute GU toxicity up to 3 months after RT are shown in **Figure 1B**. At baseline, 18%, 71%, and 11% reported grade 0, grade 1, and grade 2 GU symptoms, respectively. During RT, grade 1 and 2 GU toxicity was 65%, and 35%, respectively. At 3 months after RT, grade 1 and 2 GU toxicity was 80% and 14%, respectively. None had ≥ grade 3 GU toxicity.

**Tables 2** and **3** describe the maximal acute GI and GU toxicity reported during RT and at 3-month after RT in accordance to the presence or absence of baseline GI and GU symptoms, respectively. The presence of baseline GU symptoms was associated with a greater likelihood of experiencing acute grade 2 GU toxicity. Among patients with grade ≥ 1 GU symptoms at baseline, 44% (20 of 45) experienced grade 2 GU toxicity, in comparison with only 10% (1 of 10) in those with no baseline GU symptoms (*P* = 0.07). Similarly, among patients with baseline GI symptoms, 11% (1 of 9) patients had grade 2 GI toxicity, in comparison with none (0 of 46; 0%) in patients with no baseline GI symptoms (*P* = 0.16).

**Table 2.** Acute gastrointestinal (GI) toxicity based on the baseline GI symptoms.

**Baseline grade before RT**	**Maximum grade achieved during RT and 3 mo after RT, No. (%)**			
	**0**	**1**	**2**	**3**
0 (n = 46)	20 (43)	26 (57)	0 (0)	0 (0)
1 (n = 9)	0 (0)	8 (89)	1 (11)	0 (0)

**Abbreviation:** RT, radiotherapy.

**Table 3.** Acute genitourinary (GU) toxicity based on baseline GU symptoms.

**Baseline grade before RT**	**Maximum toxicity grade achieved during RT and 3 mo after RT, No. (%)**			
	**0**	**1**	**2**	**3**
0 (n = 10)	0 (0)	9 (90)	1 (10)	0 (0)
1 (n = 39)	0 (0)	25 (64)	14 (36)	0 (0)
2 (n = 6)	0 (0)	0 (0)	6 (100)	0 (0)

**Abbreviation:** RT, radiotherapy.

**Figure 2.**
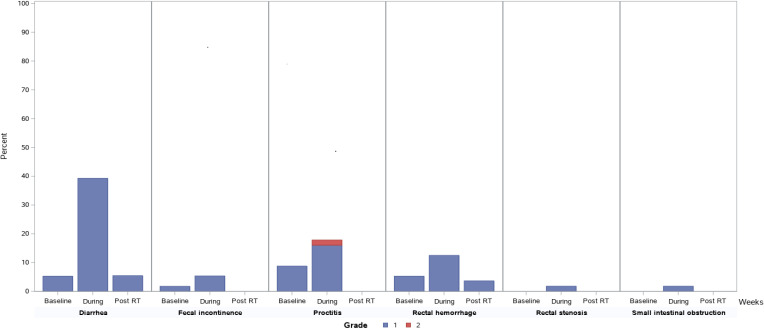
Gastrointestinal toxicity during radiotherapy (RT) and at 3 months after RT.

**Figure 3.**
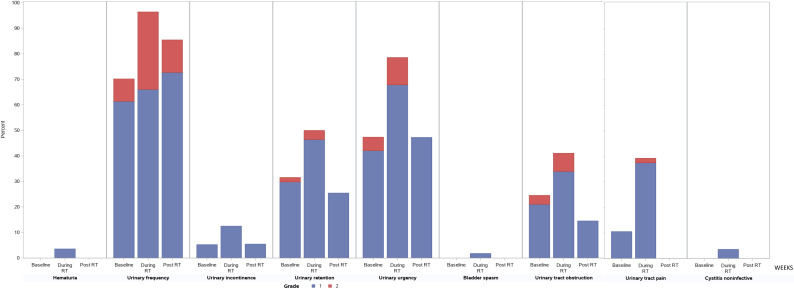
Genitourinary toxicity during radiotherapy (RT) and at 3 months after RT.

**Figures 2** and **3** depict the prevalence, severity, and types of GI and GU toxicity during RT and at 3 months after RT. **Figures 2** and **3** also include baselines to reflect the prevalence of underlying GI and GU symptoms before RT. In all the GI and GU categories, the frequency and severity of acute toxicity peaked during RT and improved at 3 months after RT. In the GI categories, the 3 most prevalent GI toxicities during RT were diarrhea, proctitis, and rectal hemorrhage. At 3 months after RT, the only reported GI toxicity was grade 1 diarrhea and grade 1 rectal hemorrhage. In the GU categories, the 3 most frequent grade 2 GU toxicities were urinary frequency, urinary urgency, and urinary tract obstruction. At 3 months, all grade 2 GU toxicities were urinary frequency.

Erectile dysfunction was very prevalent before RT, with 38% with grade 1, 31% with grade 2, and 2% with grade 3 erectile dysfunction at baseline. It was more prevalent during RT and 3 months after RT. Grade 1, 2, and 3 erectile dysfunction were 46%, 30%, and 2% , respectively, during RT, and 38%, 47%, and 0% at 3 months after RT. This higher prevalence of erectile dysfunction was expected because all patients received neoadjuvant ADT before the start of RT.

## Discussion

Our current study for high- or unfavorable intermediate-risk prostate cancer has 2 noteworthy features: (1) the application of proton beam therapy for extended CTVs covering both the regional pelvic lymph nodes and the prostate/seminal vesicles, and (2) the use of a moderately hypofractionated regimen with proton beam therapy in the setting of extended CTVs. Until now, no prospective studies, to our knowledge, have been reported evaluating the efficacy and toxicity of this strategy for the treatment of high- or unfavorable intermediate-risk prostate cancer.

Previously, we reported the dosimetric comparison between IMPT and photon-based, volumetric-modulated arc therapy (VMAT), based on 10 consecutively accrued patients [17]. Although both IMPT and VMAT plans provided adequate CTV coverage with 99% of CTVs receiving ≥ 100% of the prescription doses, IMPT significantly reduced mean doses to the small bowel, large bowel, rectum, and bladder, in comparison with VMAT. The mean dose to the small bowel was decreased by 54% with IMPT, compared with VMAT (917 cGy versus 2010 cGy, respectively). Similarly, the mean doses to the rectum, large bowel, and bladder were reduced by 36% (2347 cGy versus 3668 cGy), 29% (2155 cGy versus 3014 cGy), and 23% (2852 cGy versus 3726 cGy), respectively, with IMPT. In addition, the percentage of volumes of rectum receiving ≤ 4750 cGy, large bowel receiving ≤ 2750 cGy, small bowel receiving ≤ 3000 cGy, and bladder receiving ≤ 3750 cGy were significantly less with IMPT, largely because of the decrease in the low-to-medium dose cloud associated with VMAT.

The unresolved question is whether a large reduction in low-to-medium doses to the pelvic organs by IMPT can translate into a lower rate of GI and/or GU toxicity, in comparison with IMRT. A phase III study is required to address this important question. The outcomes of this current, single-arm, study (ClinicalTrials.gov Identifier: NCT02874014) [16] of IMPT can be the basis for launching a multi-institutional, comparative phase III study to compare IMPT with IMRT. In the realm of photon-based RT for regional pelvic nodes, it has been reported that IMRT or 3-dimensional conformal RT significantly reduces doses to the bowel, rectum, and bladder, compared with 2-dimensional RT and that those dose reductions can translate into lower rates of acute and late GI toxicity for PC [19, 20]. Ashman et al [20]. reported that 3-dimensional conformal RT resulted in a 40% relative reduction in the volume of bowel receiving 45 Gy, compared with 2-dimensional RT and that IMRT provided a further 60% relative reduction, compared with 3-dimensional conformal RT. In additional, IMRT reduced the volume of the rectum receiving 45 Gy by 90%, in comparison with 3-dimensional conformal RT. These dose reductions correlated with lower rates of acute and late GI morbidity [20]. It is also noteworthy that DVH metrics were reported to have a continuous dose effect on rectal toxicity, and the differences in the lower-dose region were more predictive of rectal toxicity than the higher-dose regions in the dose-escalation study by MD Anderson Cancer Center [21].

In our study, acute GI and GU toxicity was generally mild and self-limiting. No patients had interrupted proton beam therapy because of acute toxicity. None experienced grade 3 GI or GU toxicity. The severity and prevalence of acute GI and GU toxicity peaked during RT and then declined with time in the post-RT period, as expected. In our study, the presence of preexisting GU symptoms was associated with the greater likelihood of reporting acute GU toxicity.

Lim et al [22] reported the acute toxicity of the identical dose-fractionation regimen in a prospective study of 66 patients with high risk prostate carcinoma who were treated with photon-based, 3-D conformal RT combined with IMRT. In this study, acute grade 2 GI toxicity was 39% during RT and 6% at 3-month post-RT. Although the direct comparison of toxicity between this study and our current study is fraught with many limitations (including the use of outdated 3-D conformal RT for pelvic nodal irradiation in the study by Lim et al), our study appeared to have lower rates of grade 2 GI toxicity, 11% during RT, and 0% at 3-month post-RT. In addition, despite a higher proportion of patients with grade ≥ 1 baseline GU symptoms in our study (80% versus 29% in the study by Lim et al), our study had similar or lower rates of acute grade 2 GU toxicity during RT (35% versus 36% in the study by Lim et al) and at 3-month post-RT (12 % versus 17% in the study by Lim et al). Furthermore, none in our study had grade 3 GU toxicity, whereas grade 3 GU toxicity was 5% during RT and 3% at 3-month post-RT in the series from Lim et al.

Chuong et al [23] reported the acute toxicity of proton beam therapy on 85 patients who had irradiation to the regional pelvic lymph nodes and the prostate/seminal vesicles, using a multi-institutional prospective database. Patients were treated with 46.9 cGy (range, 39.7-56 cGy) in 25 fractions (range, 24-30) to the regional pelvic nodes, followed by a median boost dose of 30 Gy (range, 20-41.4 Gy) in 16 fractions (range, 10-24) to the prostate with or without seminal vesicles. Pelvic node metastasis was present or unknown in 22% of the cohort. No information was available for baseline GI and GU symptoms. That series reported low acute GI and GU toxicity rates, similar to ours. Acute grade 1, 2, and 3 GI toxicity rates were 16.4%, 2.4%, and 0%, respectively. Acute grade 1, 2, and 3 GU toxicity rates were 60%, 34.1%, and 0%, respectively.

The reduction in acute grade 2 GI toxicity with proton therapy is likely due to the combined effect of dose reduction to the small bowel, large bowel, and rectum, based on our dosimetric study. The dose reduction to these GI organs can translate into less-acute GI symptoms, such as diarrhea. Similarly, the reduction in acute grade ≥ 2 GU toxicity at 3 months after RT is likely largely due to the overall dose reduction to the bladder with proton beam therapy.

This study has several limitations. First, our study lacked a control group for comparison. Second, the sample size of our series was relatively small. Third, because the patients in our study received combined treatment of proton beam therapy plus ADT, the acute side effects may not be entirely due to RS and may be related, in part, to ADT.

## Conclusion

The moderately hypofractionated proton beam therapy targeting the prostate and the regional pelvic lymph nodes was generally well tolerated. No acute grade ≥ 3 GI or GU toxicity occurred. Patients with preexisting GU symptoms had a higher rate of acute grade 2 GU toxicity. A phase III study is warranted to assess whether a therapeutic ratio of RT will improve with IMPT in comparison with IMRT.

## References

[1] Zapatero A, Guerrero A, Maldonado X, Alvarez A, Gonzalez San Segundo C, Cabeza Rodriguez MA, Macias V, Pedro Olive A, Casas F, Boladeras A, de Vidales CM, Vazquez de la Torre ML, Villa S, Perez de la Haza A, Calvo FA (2015). High-dose radiotherapy with short-term or long-term androgen deprivation in localised prostate cancer (DART01/05 GICOR): a randomised, controlled, phase 3 trial. *Lancet Oncol*.

[2] Pilepich MV, Winter K, Lawton CA, Krisch RE, Wolkov HB, Movsas B, Hug EB, Asbell SO, Grignon D (2005). Androgen suppression adjuvant to definitive radiotherapy in prostate carcinoma—long-term results of phase III RTOG 85-31. *Int J Radiat Oncol Biol Phys*.

[3] Roach M, Bae K, Speight J, Wolkov HB, Rubin P, Lee RJ, Lawton C, Valicenti R, Grignon D, Pilepich MV (2008). Short-term neoadjuvant androgen deprivation therapy and external-beam radiotherapy for locally advanced prostate cancer: long-term results of RTOG 8610. *J Clin Oncol*.

[4] Lawton CAF, Lin X, Hanks GE, Lepor H, Grignon DJ, Brereton HD, Bedi M, Rosenthal SA, Zeitzer KL, Venkatesan VM, Horwitz EM, Pisansky TM, Kim H, Parliament MB, Rabinovitch R, Roach M, Kwok Y, Dignam JJ, Sandler HM (2017). Duration of androgen deprivation in locally advanced prostate cancer: long-term update of NRG oncology RTOG 9202. *Int J Radiat Oncol Biol Phys*.

[5] Bolla M, Van Tienhoven G, Warde P, Dubois JB, Mirimanoff RO, Storme G, Bernier J, Kuten A, Sternberg C, Billiet I, Torecilla JL, Pfeffer R, Cutajar CL, Van der Kwast T, Collette L (2010). External irradiation with or without long-term androgen suppression for prostate cancer with high metastatic risk: 10-year results of an EORTC randomised study. *Lancet Oncol*.

[6] Warde P, Mason M, Ding K, Kirkbride P, Brundage M, Cowan R, Gospodarowicz M, Sanders K, Kostashuk E, Swanson G, Barber J, Hiltz A, Parmar MKB, Sathya J, Anderson J, Hayter C, Hetherington J, Sydes MR, Parulekar W, NCIC CTG PR.3/MRC UK PR07 Investigators (2011). Combined androgen deprivation therapy and radiation therapy for locally advanced prostate cancer: a randomised, phase 3 trial. *Lancet*.

[7] Dasu A, Toma-Dasu I (2012). Prostate alpha/beta revisited—an analysis of clinical results from 14 168 patients. *Acta Oncol*.

[8] Miralbell R, Roberts SA, Zubizarreta E, Hendry JH (2012). Dose-fractionation sensitivity of prostate cancer deduced from radiotherapy outcomes of 5,969 patients in seven international institutional datasets: α/β = 1.4 (0.9–2.2) Gy. *Int J Radiat Oncol Biol Phys*.

[9] Fowler J, Chappell R, Ritter M (2001). Is α/β for prostate tumors really low?. *Int J Radiat Oncol Biol Phys*.

[10] Brenner DJ, Martinez AA, Edmundson GK, Mitchell C, Thames HD, Armour EP (2002). Direct evidence that prostate tumors show high sensitivity to fractionation (low α/β ratio), similar to late-responding normal tissue. *Int J Radiat Oncol Biol Phys*.

[11] Pollack A, Walker G, Horwitz EM, Price R, Feigenberg S, Konski AA, Stoyanova R, Movsas B, Greenberg RE, Uzzo RG, Ma C, Buyyounouski MK (2013). Randomized trial of hypofractionated external-beam radiotherapy for prostate cancer. *J Clin Oncol*.

[12] Catton CN, Lukka H, Gu CS, Martin JM, Supiot S, Chung PWM, Bauman GS, Bahary JP, Ahmed S, Cheung P, Tai KH, Wu JS, Parliament MB, Tsakiridis T, Corbett TB, Tang C, Dayes IS, Warde P, Craig TK, Julian JA, Levine MN (2017). Randomized trial of a hypofractionated radiation regimen for the treatment of localized prostate cancer. *J Clin Oncol*.

[13] Lee WR, Dignam JJ, Amin MB, Bruner DW, Low D, Swanson GP, Shah AB, D'Souza DP, Michalski JM, Dayes IS, Seaward SA, Hall WA, Nguyen PL, Pisansky TM, Faria SL, Chen Y, Koontz BF, Paulus R, Sandler HM (2016). Randomized phase III noninferiority study comparing two radiotherapy fractionation schedules in patients with low-risk prostate cancer. *J Clin Oncol*.

[14] Incrocci L, Wortel RC, Alemayehu WG, Aluwini S, Schimmel E, Krol S, van der Toorn PP, Jager H, Heemsbergen W, Heijmen B, Pos F (2016). Hypofractionated versus conventionally fractionated radiotherapy for patients with localised prostate cancer (HYPRO): final efficacy results from a randomised, multicentre, open-label, phase 3 trial. *Lancet Oncol*.

[15] Dearnaley D, Syndikus I, Mossop H, Khoo V, Birtle A, Bloomfield D, Graham J, Kirkbride P, Logue J, Malik Z, Money-Kyrle J, O'Sullivan JM, Panades M, Parker C, Patterson H, Scrase C, Staffurth J, Stockdale A, Tremlett J, Bidmead M, Mayles H, Naismith O, South C, Gao A, Cruickshank C, Hassan S, Pugh J, Griffin C, Hall E, CHHiP Investigators (2016). Conventional versus hypofractionated high-dose intensity-modulated radiotherapy for prostate cancer: 5-year outcomes of the randomised, non-inferiority, phase 3 CHHiP trial. *Lancet Oncol*.

[16] Prospective evaluation of hypofractionation proton beam therapy with concurrent treatment of the prostate and pelvic nodes for clinically localized, high risk or unfavorable intermediate risk prostate cancer. ClinicalTrials.gov Identifier NCT02874014.

[17] Whitaker TJ, Routman DM, Schultz H, Harmsen WS, Corbin KS, Wong WW, Choo R (2019). IMPT versus VMAT for pelvic nodal irradiation in prostate cancer: a dosimetric comparison. *Int J Part Ther*.

[18] Lawton CA, Michalski J, El-Naqa I, Buyyounouski MK, Lee WR, Menard C, O'Meara E, Rosenthal SA, Ritter M, Seider M. RTOG GU (2009). Radiation oncology specialists reach consensus on pelvic lymph node volumes for high-risk prostate cancer. *Int J Radiat Oncol Biol Phys*.

[19] Fiorino C, Alongi F, Perna L, Broggi S, Cattaneo GM, Cozzarini C, Di Muzio N, Fazio F, Calandrino R (2009). Dose-volume relationships for acute bowel toxicity in patients treated with pelvic nodal irradiation for prostate cancer. *Int J Radiat Oncol Biol Phys*.

[20] Ashman JB, Zelefsky MJ, Hunt MS, Leibel SA, Fuks Z (2005). Whole pelvic radiotherapy for prostate cancer using 3D conformal and intensity-modulated radiotherapy. *Int J Radiat Oncol Biol Phys*.

[21] Kuban DA, Tucker SL, Dong L, Starkschall G, Huang EH, Cheung MR, Lee AK, Pollack A (2008). Long-term results of the M. D. Anderson randomized dose-escalation trial for prostate cancer. *Int J Radiat Oncol Biol Phys*.

[22] Lim TS, Cheung PC, Loblaw DA, Morton G, Sixel KE, Pang G, Basran P, Zhang L, Tirona R, Szumacher E, Danjoux C, Choo R, Thomas G (2008). Hypofractionated accelerated radiotherapy using concomitant intensity-modulated radiotherapy boost technique for localized high-risk prostate cancer: acute toxicity results. *Int J Radiat Oncol Biol Phys*.

[23] Chuong MD, Hartsell W, Larson G, Tsai H, Laramore GE, Rossi CJ, Wilkinson JB, Kaiser A, Vargas C (2018). Minimal toxicity after proton beam therapy for prostate and pelvic nodal irradiation: results from the proton collaborative group REG001-09 trial. *Acta Oncol*.

